# Cytomegalovirus Infection in an Extremely Preterm Infant: A Diagnostic Challenge

**DOI:** 10.7759/cureus.111212

**Published:** 2026-06-20

**Authors:** Patricia Valiente-Rodriguez, Adriana Montealegre, Camila Merchan, Maria Rodriguez-Serpa, Nathalia Melo-Gonzalez

**Affiliations:** 1 Neonatology, Hospital Universitario San Ignacio, Bogota, COL; 2 Pediatrics, Hospital Universitario San Ignacio, Bogota, COL; 3 Medicine, Pontificia Universidad Javeriana, Bogota, COL

**Keywords:** bronchopulmonary dysplasia, combined immunodeficiency, congenital cytomegalovirus infection, extreme prematurity, neonatal cmv infection

## Abstract

Cytomegalovirus (CMV) infection in the extremely preterm infant represents a major diagnostic challenge: its clinical manifestations overlap with comorbidities inherent to prematurity, underlying immunodeficiency may suppress viral replication below detectable thresholds, and the diagnostic window for confirming congenital infection closes irreversibly at 21 days of life. An extremely preterm female infant (26 weeks, 800 g) had a neonatal course complicated by bronchopulmonary dysplasia, leukoencephalopathy, and chorioretinitis - findings that, individually, were attributable to prematurity. CMV was not tested within the 21-day diagnostic window. A plasma CMV PCR at day 44, triggered by clinical deterioration, returned negative. At 70 days of life, severe multilobar pneumonia with respiratory failure requiring high-frequency oscillatory ventilation developed without an identifiable bacterial aetiology. Targeted bronchoalveolar lavage confirmed CMV at 136,000 IU/mL, markedly dissociated from simultaneous plasma viraemia of 9,910 IU/mL. Primary combined immunodeficiency (T⁻/B⁺/NK⁺ pattern) was diagnosed simultaneously. Ganciclovir therapy achieved progressive viral load reduction. In the extremely preterm infant, CMV may remain clinically invisible behind more immediate diagnoses. A negative plasma CMV PCR does not exclude active infection in the setting of combined immunodeficiency. Severe multisystem deterioration without an identified aetiology should prompt site-directed CMV investigation and simultaneous immunological evaluation.

## Introduction

Cytomegalovirus (CMV) is the most common congenital viral infection worldwide, with an estimated prevalence of approximately one per 200 live births in high-income countries [1]. In Colombia, seroprevalence studies suggest endemic CMV transmission with high rates of maternal seropositivity, conferring substantial risk of both congenital and perinatal acquisition [2]. Definitive diagnosis of congenital CMV infection requires detection of CMV DNA in urine or saliva within the first 21 days of life; samples obtained beyond this period cannot reliably distinguish congenital from postnatal acquisition, which carries a substantially different prognosis and long-term implications [1,3]. Congenital CMV is associated with sensorineural hearing loss, neurodevelopmental delay, and chorioretinitis, whereas postnatally acquired infection in term infants is generally benign and self-limited.

The diagnostic challenge is substantially amplified in extremely preterm infants. In this population, CMV manifestations - respiratory deterioration, neurological compromise, thrombocytopaenia, hepatitis - are indistinguishable from the expected complications of prematurity itself [3]. An additional layer of complexity arises when a primary immunodeficiency coexists: impaired T-cell responses can alter viral kinetics, suppress replication below detection thresholds at conventional sampling sites, and drive compartmentalised tissue-level disease not reflected in plasma viraemia [4,5]. Combined immunodeficiency refers to a heterogeneous group of primary immune disorders characterised by defects in both T-cell and B-cell function, resulting in severe susceptibility to opportunistic infections that would otherwise be controlled by an intact immune response. CMV exerts a well-characterised tropism for neural progenitor cells, producing white matter injury, cortical migration abnormalities, progressive microcephaly, and chorioretinitis in symptomatic congenital disease [4,6,7]. At the pulmonary level, it can produce severe endobronchitis and pneumonia, particularly in immunocompromised hosts [3]. Antiviral therapy with ganciclovir or valganciclovir is indicated for symptomatic disease and has demonstrated meaningful improvements in long-term audiological and neurodevelopmental outcomes [8,9].

We present the case of an extremely preterm infant in whom the convergence of prematurity-related comorbidities and an unrecognised primary combined immunodeficiency made CMV diagnosis possible only at day 70 of life, when catastrophic respiratory deterioration without microbiological explanation finally directed clinical suspicion toward an intracellular pathogen.

## Case presentation

A female infant was born at 26 weeks of gestation (birth weight 800 g; extremely low birth weight) by caesarean section for severe maternal pre-eclampsia. Apgar scores were seven, eight, and nine at one, five, and 10 minutes. The pregnancy was unplanned with minimal antenatal care (four visits). Maternal TORCH serology was reported to be negative, but did not include CMV-specific IgM or IgG testing. A complete course of antenatal betamethasone was administered.

Initial respiratory support was nasal continuous positive airway pressure (CPAP), with endotracheal intubation required within the first 24 hours and three doses of exogenous surfactant administered. The neonatal course was characterised by prolonged mechanical ventilation, multiple failed extubation attempts, persistent multifactorial apnea, and development of bronchopulmonary dysplasia. Bronchoscopy identified severe endobronchitis and moderate-to-severe left bronchomalacia. Neurological assessment revealed poor head circumference growth and axial and appendicular hypotonia. Brain MRI demonstrated supratentorial leukoencephalopathy, subcortical microhemorrhages, and a small subacute hematoma. Ophthalmological evaluation identified retinopathy of prematurity and chorioretinitis; two sessions of retinal laser photocoagulation were performed. During the second session, a three-minute cardiorespiratory arrest occurred and was successfully reversed. Throughout this period, all findings were attributable to the comorbidities inherent to extreme prematurity, without any single finding generating specific suspicion of CMV. 

CMV virological testing was not performed during the first 21 days of life. At day 44, in the context of a first episode of clinical deterioration, plasma CMV viral load was requested for the first time and returned negative - a result that, in retrospect, was biologically plausible given the combined immunodeficiency subsequently identified.

At day 70 of life (corrected gestational age 36 weeks), the infant developed multilobar pneumonia (right upper lobe and entire left hemithorax) with severe respiratory failure, desaturations with bradycardia, and cardiorespiratory arrest episodes requiring transition to high-frequency oscillatory ventilation. Empirical coverage with meropenem, amikacin, clarithromycin, and fluconazole was initiated. Respiratory FilmArray panel was negative; blood cultures were negative at five days; urine Gram stain showed no organisms. A urine culture reported *Klebsiella pneumoniae* carbapenemase (KPC), *Escherichia coli* extended-spectrum β-lactamase (ESBL), and *Enterococcus* spp., considered probable contamination by the paediatric infectious disease team. In the absence of a microbiological explanation for the clinical severity, bronchoalveolar lavage (BAL) with targeted CMV investigation was performed.

BAL CMV PCR returned positive at 136,000 IU/mL; simultaneous plasma viral load was 9,910 IU/mL - a greater than 13-fold compartmental dissociation. Immunological workup performed the same day (07/07/2025) demonstrated a previously unrecognised primary combined cellular and humoral immunodeficiency: markedly decreased CD4+ T lymphocytes (27.9%; 791 cells/µL) and an inverted CD4/CD8 ratio of 0.8 (reference 1.7-3.9), with CD8+ T lymphocytes at 34.9% (991 cells/µL), NK cells (4.7%; 136 cells/µL) and B lymphocytes (24.84%; 720 cells/µL) within age-appropriate normal ranges; IgA <25 mg/dL and IgG <320 mg/dL, consistent with significant humoral deficit. The T⁻//NK⁺ immunophenotypic pattern is consistent with a combined immunodeficiency with predominant T-cell defect, pending genetic characterisation (Table 1).

**Table 1:** Immunological workup results CD4+ T lymphocytes were markedly decreased in both percentage and absolute count, with an inverted CD4/CD8 ratio, consistent with a predominant T-cell defect. IgA and IgG levels were below age-appropriate ranges, indicating concurrent humoral immunodeficiency. NK cells and B lymphocytes were within normal limits. Reference ranges correspond to the two- to five-month age group.

Parameter	%	Absolute count	Reference range (two to five months)	Interpretation
T lymphocytes (CD3+)	66.3	1,882 cells/µL	Not established	Normal
T lymphocytes CD4+	27.9	791 cells/µL	Not established	Markedly decreased
T lymphocytes CD8+	34.9	991 cells/µL	Not established	Normal
NK cells (CD3-/CD16+/CD56+)	4.7	136 cells/µL	2-14%/100-1,300 cells/µL	Normal
B lymphocytes (CD19+/CD20+)	24.84	720 cells/µL	14-39%/600-3,000 cells/µL	Normal
Immunoglobulin A (IgA)	N/A	< 25 mg/dL	Age-appropriate range not met	Decreased
Immunoglobulin G (IgG)	N/A	< 320 mg/dL	Age-appropriate range not met	Decreased

Intravenous ganciclovir was initiated at day 70 of life. Serial viral load monitoring demonstrated progressive decline: 337 IU/mL (day 88), 265 IU/mL (day 101), 265 IU/mL (day 105), and 117 IU/mL at the last available control (day 119), remaining detectable. Azithromycin three times per week was added as pulmonary anti-inflammatory therapy.

The brain MRI findings are illustrated in Figure 1, with arrows indicating the key areas of white matter signal abnormality and the subacute haematoma. The imaging pattern - supratentorial leukoencephalopathy with subcortical microhaemorrhages - is consistent with CMV encephalitis/leukoencephalopathy, reflecting parenchymal and white matter involvement rather than meningeal disease.

**Figure 1:**
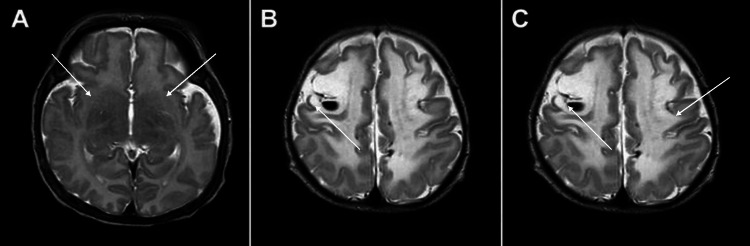
Brain MRI findings (axial T2 sequences) A) Periventricular and subcortical white matter signal abnormality consistent with supratentorial leukoencephalopathy (arrows). (B) Focal hypointense lesion in the left frontal subcortical region consistent with subacute haematoma (arrow); note associated surrounding white matter signal abnormality. (C) Diffuse supratentorial white matter hyperintensity with subcortical involvement, consistent with leukoencephalopathy (arrows).

At the time of this report, the infant is one year of age and has been discharged from the hospital. Follow-up has demonstrated global neurodevelopmental delay, with developmental achievements currently limited to visual tracking and head control. She remains under multidisciplinary outpatient surveillance, including neurology, ophthalmology, immunology, and infectious disease. This outcome is consistent with the expected neurodevelopmental trajectory of symptomatic congenital CMV disease complicated by extreme prematurity and underlying combined immunodeficiency.

## Discussion

This case illustrates why CMV diagnosis in the extremely preterm infant is a genuine clinical challenge, not simply a consequence of insufficient surveillance. Three factors converged to keep the infection unrecognised for 70 days: clinical overlap with prematurity-related pathology, a negative plasma viral load attributable to an unrecognised immunodeficiency, and the absence of an early finding specific enough to direct diagnosis before catastrophic deterioration occurred.

The first and most important factor was clinical overlap. In a 26-week preterm infant, prolonged mechanical ventilation, bronchopulmonary dysplasia, apnoeas, leukoencephalopathy, subcortical microhaemorrhages, and chorioretinitis are findings explicable by extreme prematurity and its direct complications. CMV shares this clinical repertoire almost completely: its tropism for neural progenitor cells generates white matter lesions indistinguishable from periventricular leukomalacia of prematurity. The MRI pattern observed in this case - supratentorial leukoencephalopathy with subcortical microhaemorrhages - is consistent with CMV encephalitis/leukoencephalopathy rather than meningitis, reflecting white matter and parenchymal involvement rather than meningeal disease [4]. Its capacity to produce endobronchitis and bronchomalacia can be mistaken for sequelae of prolonged mechanical ventilation [3]. No isolated finding was sufficiently specific to prioritise CMV as a differential diagnosis. In retrospect, however, the combination of progressive microcephaly, supratentorial leukoencephalopathy with subcortical microhaemorrhages, and chorioretinitis is more consistent with symptomatic congenital CMV disease than with a postnatally acquired form, which in premature neonates typically presents as a sepsis-like illness without the structural neurological signature observed here [7,10,11].

The second factor was the negative plasma viral load at day 44. This result was biologically plausible: the combined immunodeficiency identified weeks later, with markedly decreased CD4+ T lymphocytes and an inverted CD4/CD8 ratio, alongside an immunoglobulin deficit, was likely already present. We hypothesise that the impaired T-cell response may have suppressed viral replication below detection thresholds at conventional sampling sites while permitting uncontrolled compartmentalised replication in others, particularly at mucosal surfaces and within lung parenchyma [4,5]; the greater than 13-fold dissociation between BAL (136,000 IU/mL) and plasma viral load (9,910 IU/mL) observed in this case is consistent with this mechanism, though direct proof is not available from the case data alone [12]. This finding challenges the practice of relying on plasma CMV PCR as the primary monitoring tool in immunocompromised neonates and supports a lower threshold for site-directed sampling when clinical deterioration remains unexplained. The Colombian epidemiological context is also relevant: Rico et al. documented high CMV seroprevalence among Colombian mothers and infants, conferring a substantially higher pretest probability of CMV exposure than in low-seroprevalence populations where universal neonatal screening has been implemented - a context that strengthens the argument for maintaining high clinical suspicion [2].

The third factor was the absence of an early, specific finding to anchor CMV suspicion. It was the catastrophic respiratory deterioration - multilobar pneumonia with severe respiratory failure, high-frequency oscillatory ventilation, and cardiorespiratory arrests, without bacterial or fungal aetiology after exhaustive investigation - that finally directed suspicion toward an intracellular pathogen. Bacterial infection was excluded by negative blood cultures at five days, and negative urine Gram stain; fungal aetiology was considered unlikely given the absence of response to empirical fluconazole and negative cultures; viral respiratory pathogens were excluded by a negative FilmArray respiratory panel. The indication of BAL CMV PCR was the diagnostic turning point. This pattern underscores that in extremely preterm infants with immunodeficiency, CMV should be actively reconsidered in the differential diagnosis of severe unexplained multisystem deterioration, regardless of prior negative results [1,3].

The simultaneous identification of primary combined immunodeficiency adds a clinically important dimension. The CD4-low/B⁺/NK⁺ immunophenotype - markedly decreased CD4+ T lymphocytes with an inverted CD4/CD8 ratio of 0.8, alongside B lymphocytes and NK cells within normal ranges - is consistent with a combined immunodeficiency with predominant T-cell defect, representing a partial SCID variant of aetiology pending genetic characterisation. This condition not only explains the severity and progression of CMV infection but also the persistence of detectable viral load under antiviral therapy [4,5]. The coexistence of both diagnoses, discovered simultaneously, constitutes a high-complexity scenario requiring long-term multidisciplinary follow-up.

From a therapeutic standpoint, intravenous ganciclovir achieved progressive and sustained viraemia reduction. Persistence of detectable viral load at the last available control is expected in the context of severe combined immunodeficiency and should not be interpreted as treatment failure. Current guidelines recommend oral valganciclovir for six months as standard of care for confirmed symptomatic congenital CMV; in diagnostically ambiguous cases with severe disease, decisions should be individualised according to clinical and virological response [8,9].

## Conclusions

CMV diagnosis in the extremely preterm infant is a genuine clinical challenge rooted in the overlap of its manifestations with prematurity-related pathology, the possibility of negative virological results in the presence of underlying immunodeficiency, and the absence of pathognomonic early findings. In this context, delayed diagnosis does not reflect insufficient clinical vigilance but the intrinsic difficulty of suspecting a specific pathogen when its presentation is indistinguishable from more immediate diagnoses. A negative plasma CMV PCR does not exclude active infection when combined immunodeficiency is present or suspected; compartmentalised pulmonary replication may be the dominant disease pattern while plasma viraemia remains low or undetectable. The practical message of this case is that in extremely preterm infants with severe multisystem deterioration without an identified aetiology - particularly when severity exceeds what is attributable to prematurity alone - CMV should be actively reconsidered through site-directed sampling from the anatomical compartment of greatest compromise, accompanied by systematic immunological evaluation. The neurodevelopmental outcome observed in this case - global delay with achievements limited to visual tracking and head control at one year of age - underscores the devastating consequences of delayed diagnosis. Early recognition and timely initiation of antiviral therapy remain the most effective tools available to limit end-organ damage and improve long-term neurodevelopmental outcomes in this population.
